# Endocardial pacing in a single‐ventricle patient with tricuspid atresia—a case report

**DOI:** 10.1002/ccr3.7945

**Published:** 2023-09-21

**Authors:** Ahmad Yamini‐Sharif, Ramin Yaghoobian, Homa Ghaderian, Najme‐Sadat Moosavi

**Affiliations:** ^1^ Tehran Heart Center, Cardiovascular Diseases Research Institute, Tehran University of Medical Sciences Tehran Iran

**Keywords:** congenital heart disease, epicardial pacing, perirenal fluid, transvenous pacing, tricuspid atresia, univentricular hearts

## Abstract

**Key Clinical Message:**

The use of endocardial pacing in patients with univentricular hearts and intracardiac shunts is limited, primarily due to the increased risk of thromboembolism. However, when accompanied by proper long‐term anticoagulation therapy, it may be safer than epicardial interventions in selected patients at high risk for surgery.

**Abstract:**

We report transvenous endocardial pacing through the atrial septal defect in a patient with tricuspid atresia, transposition of the great arteries, severe pulmonary hypertension, and complete heart block. This study is among the first reported cases using this pacing method in a patient with a univentricular heart and intracardiac shunt.

## Graphical abstract

Dual‐Chamber Endocardial Pacing for a Univentricular Heart with Tricuspid Atresia.

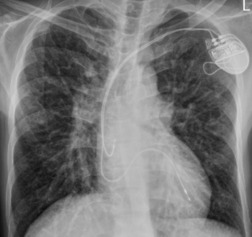

## 1 INTRODUCTION

Multiple congenital heart diseases have progressive conduction disorders, either intrinsic or developed through reparative surgeries.^1^ In particular, univentricular hearts are more associated with bradyarrhythmias and complete heart block. Many of these patients need a permanent pacemaker.^2^ In univentricular hearts, epicardial pacing has been the first option because of the limited venous access. Also, in the presence of intracardiac shunts, it is the preferred method since there is an increased risk of systemic thromboembolism with the endocardial route.^1^, ^2^, ^3^ However, epicardial leads have considerable disadvantages. One of them is that they have higher chronic pacing thresholds and reduced generator longevity, although this may be improved by using steroid‐eluting epicardial leads. Another problem is invasive surgeries used for implanting these leads.^4^, ^5^ The present report explains the implantation of a permanent endocardial pacemaker in an adult patient with tricuspid atresia type IIc with severe pulmonary hypertension and complete heart block.

## 2 CASE PRESENTATION

**FIGURE 1:**
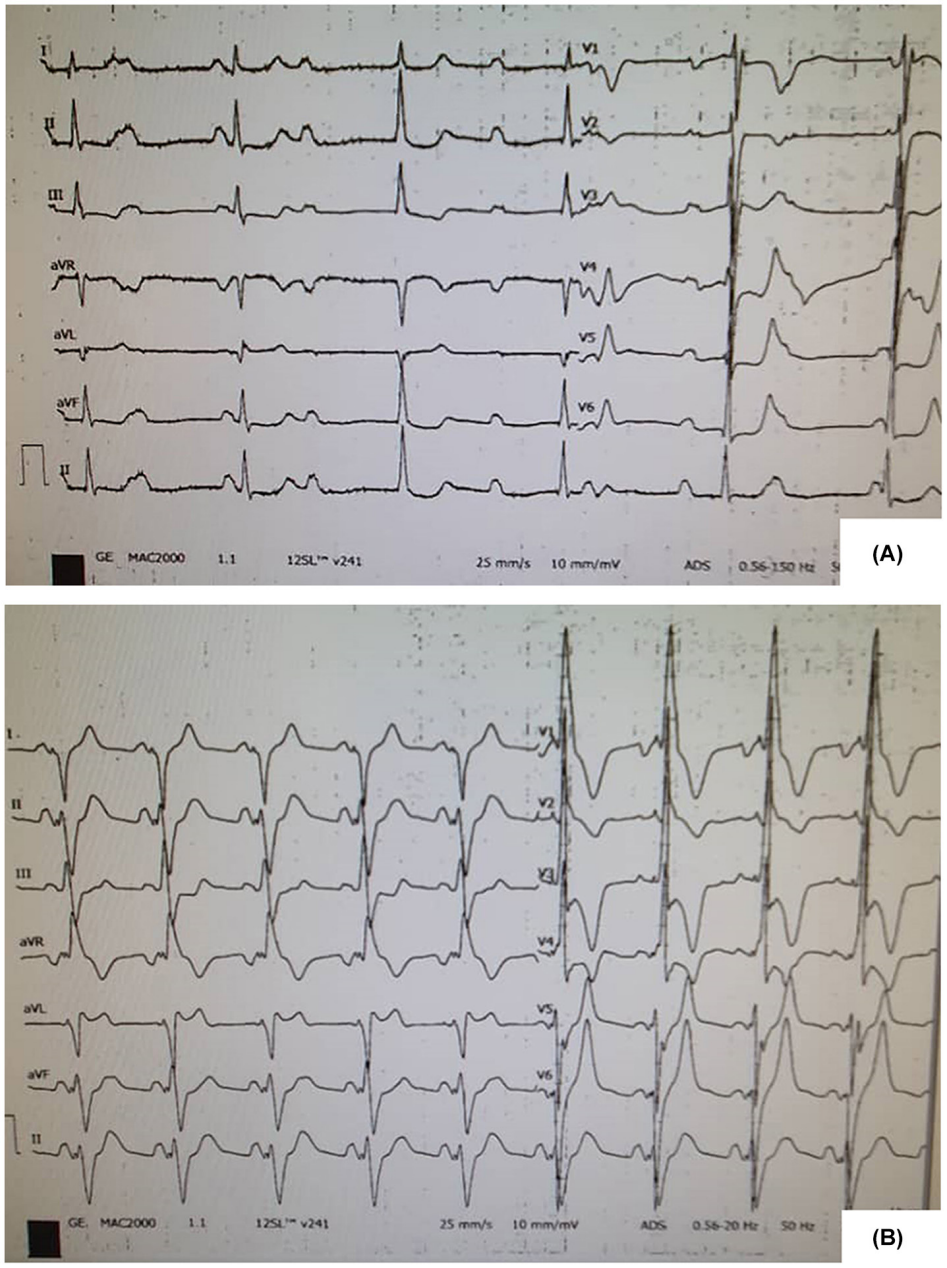
The 12‐lead electrocardiogram of the patient before and after dual chamber pacemaker implantation. (A) Complete heart block with a right bundle branch block. (B) Pace rhythm.

**FIGURE 2:**
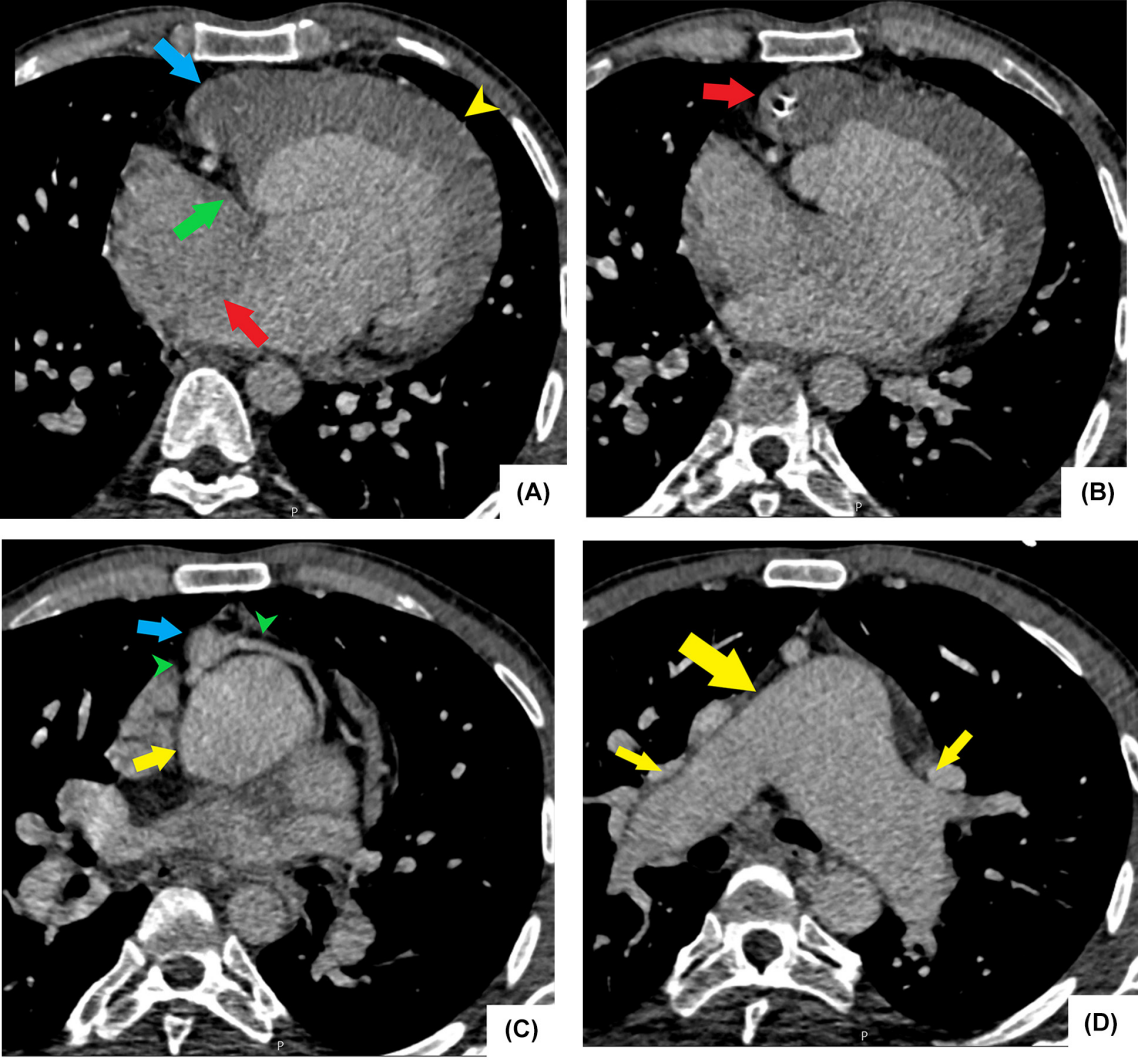
Cardiothoracic CT scan. Axial images show (A) four‐chamber image shows atresia of the tricuspid valve (green arrow) and hypoplastic right ventricle (blue arrow). There is a large atrial septal defect (red arrow), and the left ventricle is enlarged and hypertrophic (yellow arrowhead). (B) Atretic and calcified aortic valve (red arrow) originating from hypoplastic RV (L‐TGA). (C) Hypoplastic ascending aorta (blue arrow) located anterior to the main pulmonary artery (yellow arrow). Both coronary arteries (green arrows) have normal anatomy. (D) Dilated central pulmonary arteries (yellow arrows) indicate pulmonary hypertension.

**FIGURE 3:**
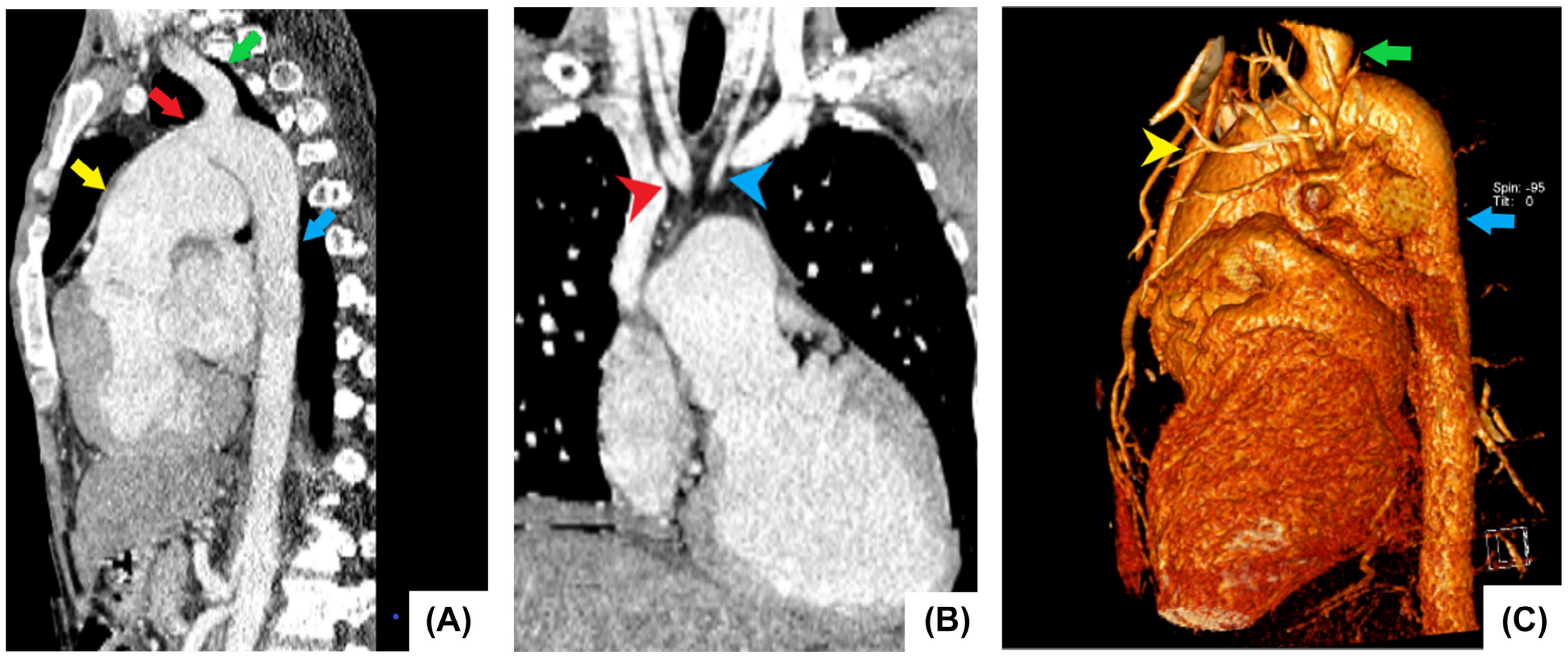
Cardiothoracic CT scan. Reconstructed images show interrupted aortic arch type B, (A) patent ductus arteriosus (red arrow) provides blood flow from the pulmonary artery (yellow arrow) to the left subclavian (green arrow) and descending thoracic arteries (blue arrow). (B) Brachiocephalic (red arrowhead) and left common carotid (blue arrowhead) arteries arise from the proximal aortic arch. (C) In the 3D image, there is an apparent interruption of the aortic arch between the hypoplastic ascending aorta (yellow arrowhead) and descending aorta (blue arrow) proximal to the left subclavian artery (green arrow).

**FIGURE 4:**
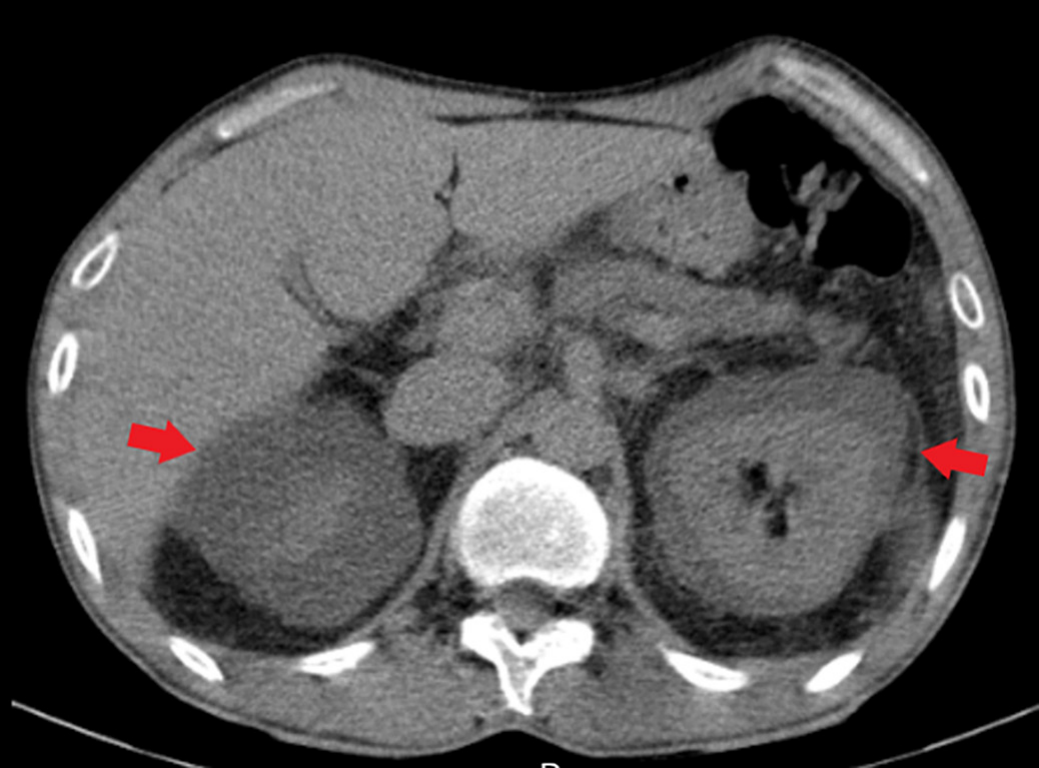
Non‐contrast abdominal CT scan. Axial image shows bilateral perirenal fluid accumulation (red arrows).

A 42‐year‐old man from Iraq with frequent episodes of syncope since 1 month ago was admitted to the Tehran heart center hospital. He had a history of tricuspid atresia type IIc without reparative surgery. On his admission, the patient had fatigue. On physical examination, he had nail clubbing and cyanosis with an O_2_ saturation of 75%. His electrocardiogram revealed a complete heart block with a right bundle branch block (Figure 1). Echocardiography showed tricuspid atresia with transposition of the great arteries (TGA type), severe pulmonary hypertension (mean of PAP: 85 mmHg), and dominant left ventricle with LVEF: 35%. Cardiothoracic CT was done to evaluate better the cardiac structure, which revealed a large pulmonary trunk, hypoplastic ascending aorta, aortic arch with large PDA, and interrupted aorta. Detailed findings of the cardiothoracic CT scan are shown in Figures 2 and 3. In abdominal CT, an accidental finding was bilateral perirenal fluid accumulation (Figure 4).

**FIGURE 5:**
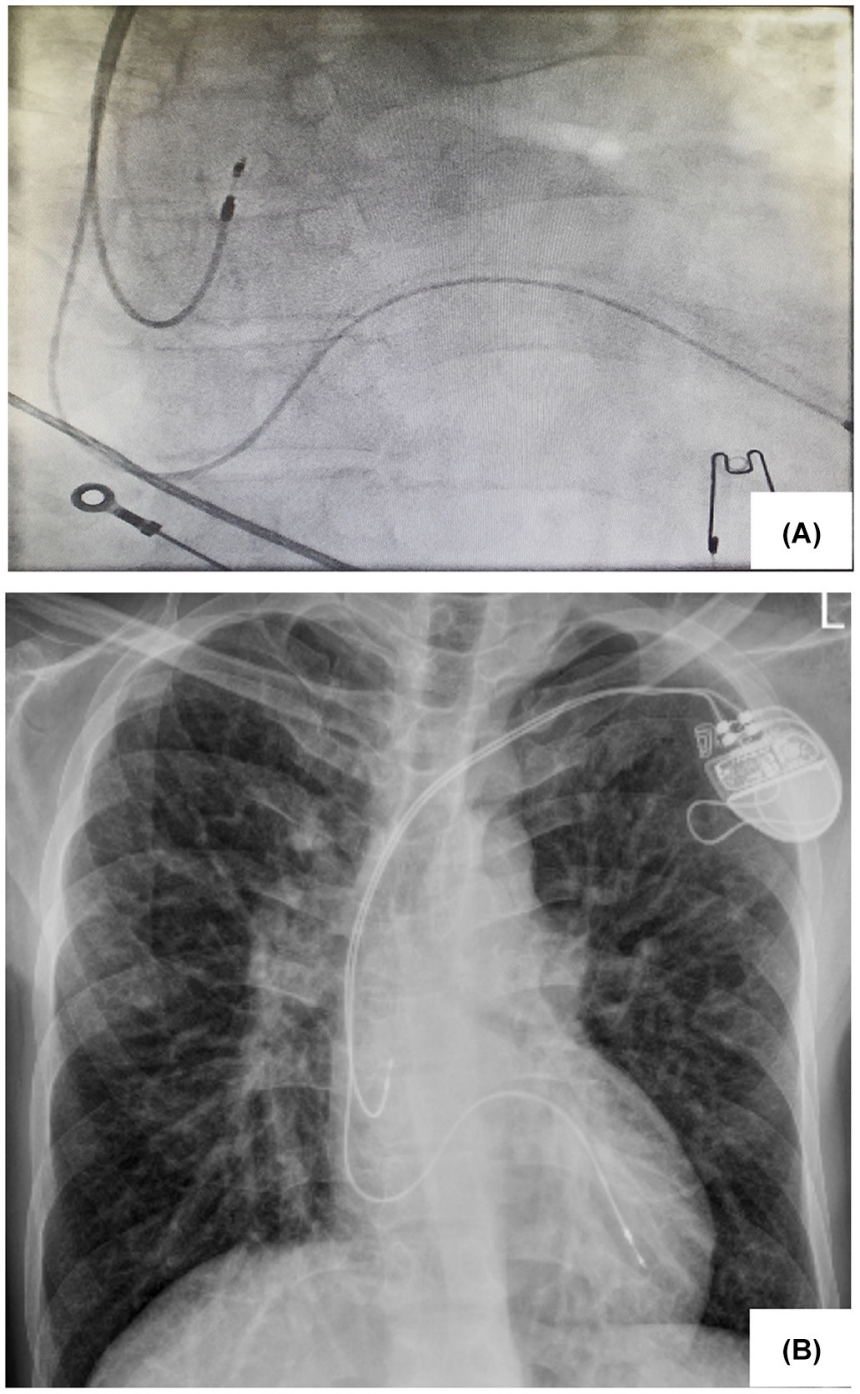
Final position of the leads, (A) anteroposterior (AP) fluoroscopic view. (B) Posteroanterior (PA) chest X‐ray.

The risk of the surgery for the epicardial lead placement was high due to pulmonary hypertension; on the other hand, the transvenous pacing would increase the risk of systemic thromboembolism. We discussed the risk associated with each procedure with the patient and recommended the endocardial route, which he accepted. Thus, endocardial pacemaker implantation was scheduled for the patient. After prep and drape, local anesthesia and light sedation were conducted. The left subclavian vein access was gained, and a dual‐chamber rate‐modulated permanent pacemaker was implanted using a peel‐away introducer. Atrial lead was inserted in the right atrial auricle. Ventricular lead was advanced through the atrial septal defect and mitral valve and was placed in the common ventricle. The final position of the leads is indicated in Figure 5. The patient was on heparin during the procedure, and lifelong warfarin was initiated for the patient 24 h after the surgery. During the first 3 months, the patient was evaluated every 2 weeks, and the INR was monitored to be in the 2–3 range. From then the patient received the follow‐up monthly. On 2 year follow‐up, the patient was well and had no complaints. The pacemaker was in the proper position without clot formation. The post‐pacing electrocardiogram is shown in Figure 1.

## 3 DISCUSSION

Epicardial pacing has been a regular option for patients with complex congenital heart disease. Epicardial leads may be placed during cardiac surgery for underlying congenital heart disease with a plan to replace them in the future with transvenous leads.^4^ Endocardial pacing of the ventricle for patients with Fontan circulation was impractical due to the lack of transvenous access to the ventricle. With the advances in pacing techniques, ventricular pacing in these patients is possible. Hsieh et al. reported pacing the ventricle in a univentricular patient with Fontan circulation and complete atrioventricular (AV) block via the coronary sinus.^6^ In another study, DeWitt et al. reported successfully placing an endocardial ventricular pacing lead through a trans‐Fontan‐baffle puncture in a single ventricle patient.^7^ These techniques may be good alternatives to high‐risk epicardial pacing methods in this group of patients.

Endocardial ventricular pacing is technically possible in patients with univentricular hearts without previous reconstructive surgery, but the higher risk for thromboembolism limits this procedure.^2^ In a cohort study of 202 patients, transvenous pacemakers increased the risk of systemic thromboembolism in patients with intracardiac shunt by >2‐fold, and anticoagulant therapy with warfarin or aspirin did not reduce the risk.^3^ However, not many studies confirmed the inefficiency of anticoagulation therapy in patients with intracardiac shunts and transvenous endocardial pacing.

Our report explains a technique including the insertion of an endocardial ventricular lead through the intra‐atrial septum. This method is similar to the one employed in patients with normal cardiac anatomy who undergo transseptal puncture. In a similar study, Scott et al. reported endocardial ventricular pacing in a univentricular patient with tricuspid atresia and intracardiac shunt using the same method we used. The patient had no thromboembolic events or complications related to the procedure at the 1‐year follow‐up.^8^

Therefore, transvenous endocardial pacing may be the better option for high‐risk patients for surgery. This case was a candidate for a Fontan procedure with epicardial lead placement, but this was a high‐risk procedure considering his chronic high pulmonary artery pressure. As a result, we deemed that transvenous lead placement with a tight anticoagulant regimen has a lower risk.

Only a few studies have compared the outcomes of dual‐chamber pacing versus single‐chamber ventricular pacing in patients with single ventricle physiology. Nevertheless, dual‐chamber pacemakers are generally considered in these patients because of AV synchrony, which enhances cardiac output and the capacity to interfere if atrial arrhythmia or sick sinus syndrome occurs.^9^

The bilateral perirenal fluid accumulation was a rare finding in the present case. It has been reported in cases with intracardiac shunt and pulmonary hypertension.^10^, ^11^ Pentimone et al. proposed that the underlying mechanism is that pulmonary hypertension increases local hydrostatic pressure in the perirenal veins, leading to fluid leakage to the renal subcapsular space. In their case, repeated phlebotomies lowered hematocrit and reduced perirenal fluid significantly.^10^

In conclusion, endocardial pacing, in the presence of intracardiac shunts, has the risk of systemic thromboembolism. Nevertheless, when accompanied by proper anticoagulation therapy and tight control of INR at 2–3, it may be a safer alternative for epicardial interventions in patients at high risk for surgery.

## AUTHOR CONTRIBUTIONS

**Ahmad Yamini‐Sharif:** Conceptualization; supervision; writing – review and editing. **Ramin Yaghoobian:** Writing – original draft; writing – review and editing. **Homa Ghaderian:** Supervision; writing – review and editing. **Najme‐Sadat Moosavi:** Writing – review and editing.

## CONFLICT OF INTEREST STATEMENT

The authors declare that they have no conflicts of interest.

## ETHICS STATEMENT

Ethical approval for this case report was not required.

## CONSENT

Written informed consent was obtained from the patient for the publication of this report.

## Data Availability

Data sharing is not applicable to this article as no new data were created or analyzed in this study.
